# Physical activity as an exogenous risk factor for amyotrophic lateral sclerosis: a review of the evidence

**DOI:** 10.1093/brain/awac470

**Published:** 2023-02-22

**Authors:** Laura Chapman, Johnathan Cooper-Knock, Pamela J Shaw

**Affiliations:** Sheffield Institute for Translational Neuroscience (SITraN), University of Sheffield, Sheffield, UK; Sheffield Institute for Translational Neuroscience (SITraN), University of Sheffield, Sheffield, UK; Sheffield Institute for Translational Neuroscience (SITraN), University of Sheffield, Sheffield, UK

**Keywords:** amyotrophic lateral sclerosis, motor neuron disease, strenuous physical activity, environmental risk factor

## Abstract

Amyotrophic lateral sclerosis (ALS) is a rapidly progressive and fatal neurodegenerative disorder. The only established epidemiological risk factors for ALS are male sex and increasing age. The role of physical activity has been debated as an environmental risk factor. Over the last decade multiple studies have attempted to delineate the architecture of ALS. These have not yet established definite risk factors, often due to low-powered studies, lack of focus on at-risk genotypes and sub-optimal methodology.

We have conducted a review of all the studies published between 2009 and December 2021. The free text search terms were [(motor neuron disease) OR (MND) OR (Amyotrophic Lateral Sclerosis) OR (ALS)] AND [(Exercise) or (Physical Activity) or (PA) or (sport)]. We identified common themes, for example soccer, head injury and the physiological mechanisms that differ in ALS patients. We have analysed the relevant, available studies (*n* = 93), highlighting the underlying reasons for any reported discrepancies.

Overall, we have found that the more highly powered studies using validated exposure methodologies, linked strenuous, anaerobic physical activity as a risk factor for ALS. Future large-scale studies focusing on specific at-risk genotypes and physical activity should be conducted to confirm this finding. This will strengthen the evidence already surrounding strenuous physical activity as an environmental risk factor for ALS and allow advice to be given to at-risk family members. Increasing our understanding of the genetic–environmental interactions in the pathophysiology of ALS will allow for the possibility of developing preventative therapeutic approaches.

## Teaser

Chapman *et al.* review published evidence evaluating the link between strenuous physical activity and the development of amyotrophic lateral sclerosis. The emerging consensus supports this environmental exposure as a risk factor for ALS and suggests that underlying genetic susceptibility factors are likely to be important.

## Introduction

Amyotrophic lateral sclerosis (ALS) is the most common subtype of motor neuron disease (MND), and the terms are often used interchangeably. It is a rapidly progressive and fatal disorder, with life expectancy commonly only 2–3 years from symptom onset.^1,2^ The upper and lower motor neurons (MNs) degenerate causing progressive failure of the neuromuscular system, affecting the function of the upper and lower limbs, as well as the bulbar and respiratory muscles. The burden of ALS globally has increased substantially from 1990–2016, with a current estimated life-time risk of approximately 1 in 300.^3,4^

The aetiology of ALS is considered to be due to a combination of risk-genotypes that interact with environmental factors over time, accelerating the neurodegenerative cascade.^5–8^ As such, ALS is an archetypal complex disease. Heritability studies suggest that ∼60% of ALS risk is genetically determined, and the remainder is environmental.^6^ Many of the genetic mutations linked to ALS, even those which are highly penetrant, are present for more than 50 years before disease onset. Indeed, the late age of onset of ALS points to a multistep process, in which genetic risk factors are penetrant only in the presence of additional environmental ‘hits’.^9^ The most common mutation linked to ALS is an intronic G4C2-repeat expansion of C9ORF72, which affects ∼10% of all ALS patients. The penetrance and severity of C9ORF72-ALS is markedly variable, which is consistent with interacting environmental factors.^10,11^ To date, the only confirmed epidemiological risk factors for ALS are male sex and increasing age, with onset most commonly between 60 and 75 years of age.^12,13^ Over the last decade, multiple reports have been released attempting to delineate the environmental risk factors predisposing to the development of ALS, however these have often been underpowered and have not led to significant advances in the field. The identification of a specific gene–environmental interaction could play an important role in disease prevention and a future personalized medicine approach. This review focuses on physical activity (PA) as a risk factor, and its interaction with the commonest known genetic risk factor, mutations in the *C9orf72* gene, as a monogenic risk factor. There are currently no clear data on the link between polygenic genetic risk factors and PA as an exogenous environmental risk factor.

The role of PA in the aetiology of ALS has been debated over several decades. ALS has been commonly documented in high profile athletes. This group are reported to have a higher incidence and lower age of onset of ALS, leading to the hypothesis that strenuous, repetitive exercise may represent an environmental risk factor.^14^ However, various studies have failed to confirm a link between PA and ALS.^15–19^ These conflicting conclusions may be explained by small study numbers, selection bias, no specific focus on risk-genotypes and the use of non-validated methods for quantification of historical levels of PA exposure.^20^ The controversy surrounding this hypothesis also relates to the benefits that regular exercise provides, for example for the cardiovascular system, leading to a potential survival bias.^21^ Most athletes and physically active individuals clearly do not develop ALS and it could be argued that the benefits of physical exercise outweigh the small potential risk of the development of ALS. However, a deeper understanding of this potential genetic–environmental interaction would allow for more personalized, focused advice for patients and family members and potentially could lead to the development of preventative strategies.

Previous reviews focusing on PA and ALS concluded that there was insufficient evidence to make a definite conclusion on PA as a risk factor for ALS.^20,22^ The present review aims to evaluate the recent evidence emerging since 2009, to provide a more conclusive answer as to whether or not PA is a factor in aetiology of ALS. An outline of each study’s main findings can be seen in Table 1 and Supplementary Table 1.

**Table 1:** A summary of the reports 2009–21, investigating whether PA is an environmental risk factor for ALS

Publications	Summary conclusion: positive association
Daneshvar *et al*.^23^	Significantly higher incidence and mortality with ALS among NFL players than the US population. Also a direct link with increased length of NFL career.
Raymond *et al*.^24^	Participants involved in vigorous PA ≥3 times per week more likely to develop ALS and at a younger age.
Gamez *et al*.^25^	Mean age of onset of ALS was younger in Spanish professional/semi-professional football players by 23.7 years compared to the general European population.
Russell *et al*.^26^	Neurodegenerative disease risk in soccer players was highest in defenders and players that had played for >15 years.
Julian *et al*.^27^	MR evidence supported a relationship between genetic liability to frequent strenuous leisure-time exercise and ALS. Transcriptomic analysis showed that genes altered in response to acute exercise are enriched with known ALS risk genes. A positive relationship found between age of onset of G4C2-repeat expansion *C9orf72*-ALS and strenuous, leisure-time exercise.
Westeneng *et al*.^28^	Daily energy intake at symptom onset in C9-positive group and C9-negative group higher versus controls. Concluded that exercise increases the risk of ALS.
Canosa *et al*.^29^	^18^F-FDG-PET demonstrated that in a group of ALS patients expressing the same level of disability, hypermetabolism was found in the subgroup that did not exercise, suggesting that this hypermetabolic group could potentially adapt better to the neurodegenerative process.
Fang *et al*. ^30^	Examining a large cohort of cross-country skiers, as a high endurance exercise, the authors reported that the fastest skiers had a 4-fold increased risk of developing ALS. Participants who had been involved in over four races compared to a single race were also at increased risk.
Harwood *et al*.^31^	The use of the HAPAQ questionnaire showed that the level of total PA throughout adulthood is significantly associated with the risk of developing ALS.
Pasquinelli *et al*.^32^	ALS patients with Gly482Ser allelic variant in *PGC1α* show increased exercise-related oxidative stress. ALS participants had significantly increased advanced oxidation protein products, decreased ferric reducing ability and thiol groups in comparison to controls.
Lehman *et al*.^33^	Neurodegenerative mortality from ALS and Alzheimer’s disease was increased 4-fold in professional footballers’ versus the general US population.
Turner *et al*.^34^	The was concordance for side of onset and handedness in upper limb-onset ALS patients, implying a higher exercise demand can trigger the site of onset of ALS.
**Summary conclusion: negative association**	
Korner *et al*.^35^	In a cohort study, ALS patients did not report increased PA at leisure or occupation versus controls.
Venkataramni *et al*.^19^	There was no statistically significant difference in the risk of long-term all-cause mortality among career NFL players versus NFL replacement players who participated in the NFL during a three-game league-wide player strike in 1987.
Janssen *et al*.^18^	American varsity high-school football players had an increased risk of medically documented trauma, but did not have increased risk of neurodegenerative disease overall.
Savica *et al*.^16^	In high school football players, there was no increased risk of Alzheimer’s dementia, Parkinson’s disease or ALS.

See Supplementary Table 1 for further critical analysis. HAPAQ = historical adulthood physical activity questionnaire; NFL = National Football League.

## Materials and methods

### Search strategy and selection criteria

**Figure 1:**
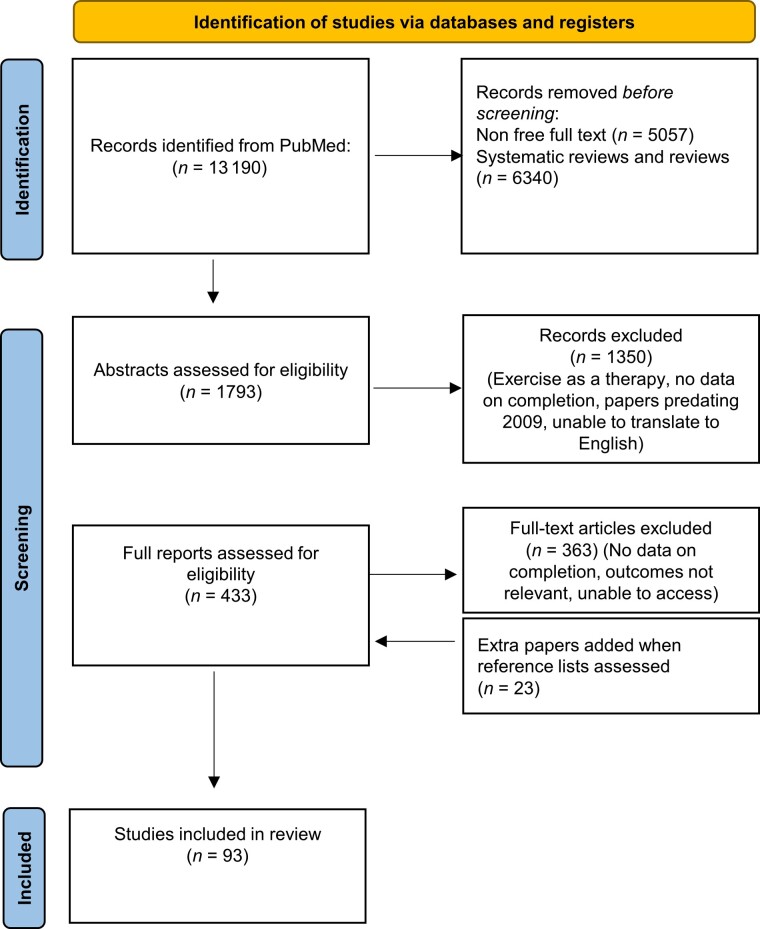
**PRISMA flow diagram.** PRISMA flow diagram detailing the bibliographical searches carried out, including the number of papers screened and the reasons for any publications which were excluded from the analysis.^36^

The papers for this review were identified by searching PubMed between 2009 and December 2021. The reference lists of these articles were searched to identify further relevant publications. The free text search terms were [(motor neuron disease) OR (MND) OR (amyotrophic lateral sclerosis) OR (ALS)] AND [(exercise) or (physical activity) or (PA) or (sport)]. The inclusion criteria included papers published since 2009 and in English language or that could be translated. The exclusion criteria included: papers focusing on paediatric patients; inability to access the full article text; papers which did not report clear outcomes; papers which could not be translated into the English language; papers discussing PA as a therapeutic measure rather than a risk factor; and systematic reviews. The final reference list was generated on the basis of significance to the topics covered in this review. The PRISMA flow diagram for this review is shown in Fig. 1.^36^

## Results

### Quantification of unselected physical activity and ALS

PA has previously been defined as activity that is ‘planned, structured and repetitive’ with an immediate or long-term goal to improve or maintain physical fitness.^37^ PA consists of either aerobic or anaerobic activity, with the involvement of specialized muscle fibres and motor neurons. Aerobic activity involves large muscle groups and can be maintained continuously. These muscles rely on oxygen to generate energy in the form of adenosine triphosphate (ATP) from amino acids, carbohydrates, and fatty acids. Aerobic PA or capacity relies on the capacity of the cardiorespiratory system to supply oxygen and the skeletal muscles capacity to utilize this oxygen. Aerobic capacity can be measured by peak oxygen consumption (VO_2_). Examples of aerobic PA are cycling, dancing, and long distance running and walking.^38^ Anaerobic PA involves high intensity, short duration exercise and uses the fast twitch muscles. Examples include sprinting or high-intensity interval training. The muscles experience a lack oxygen; therefore, the cells use glycolysis and fermentation to produce ATP. This produces less ATP than aerobic PA and causes an increase in lactic acid.^38^ In ALS it is this fast twitch (type IIb) anaerobic activity, i.e. vigorous activity, which is considered to be the predominant risk factor.^27,38^ Many studies have focused on different types of PA and their potential as an exogenous risk factor for ALS. However, the majority have not specifically divided the PA into aerobic and anaerobic activities, and this is likely to have a large impact and may account for some of the variability in conclusions reached in different reports.

Harwood *et al*.^31^ conducted a case-control study focusing on the link between PA and ALS. They measured PA exposure during adult life using the HAPAQ. This questionnaire, developed in collaboration with the Medical Research Council Epidemiology Unit at Cambridge University, was validated in the separate Ely cohort of participants. HAPAQ was the first questionnaire instrument to measure historical levels of PA validated against repeated historical objective activity measurements.^39^ Harwood and colleagues^31^ used HAPAQ to quantify PA exposure in a regional cohort of 175 newly diagnosed ALS cases and 317 age and gender population matched controls. The main finding of this study was that participation in an extra 10 kJ/kg of daily PA (equivalent to ∼45 min of brisk walking) was consistently associated with an increased risk of ALS [odds ratio (OR) 1.47, 95% confidence interval (CI) 1.10–1.97]. Positive associations were reported for all domains of PA, with the strongest associations for individuals reporting higher levels of vigorous and exercise-related PA throughout adulthood.^31^ The data were collected retrospectively, increasing the risk of recall bias and over-estimation of activity levels, due to the social desirability of being more physically active. The nature and relative rarity of ALS make it difficult to avoid a relatively small cohort. Advantages of this study included blinding of the participants to the research hypothesis, therefore reducing recall bias. The careful selection of control participants and the use of a validated questionnaire were additional advantages.

Despite being validated, there are still difficulties with a questionnaire-based approach to quantify individual levels of PA, due to personal bias. Another way of measuring PA could be via wearable devices. However, a recent review looked at the accuracy of wrist and arm-worn monitors in estimating energy expenditure and found that they differed depending on activity type.^40^ Therefore, the use and accuracy of wearable devices needs to be studied further. In future studies, wearable devices could be used alongside the HAPAQ questionnaire to increase reliability and decrease bias.

In the report of Harwood *et al*.,^31^ HAPAQ was conducted face-to-face, with life calendars of significant events to aid recall of historical levels of PA. This stringent approach increases the reliability of the study and allows us to draw the conclusion that PA is positively associated with the development of ALS, at least in this population of patients in the North of England.

A disadvantage of the HAPAQ is that it cannot easily be applied to thousands of patients because it is relatively labour-intensive. Another recent study from Julian *et al.*^27^ set out to use Mendelian randomization (MR) as a means of overcoming limitations due to sample size. MR is a method whereby genetic variation within a population established at conception and which determines genetic liability for exposure to a risk factor of interest, serves as an instrument to study the effect of environmental modifiers on an outcome such as the risk of a specific disease, in this case ALS. It allows PA and ALS to be quantified in separate cohorts which removes the requirement for two measurements per patient, which can create a bottleneck for recruitment. In addition, MR does not suffer from problems with selection and recall bias that plague observational studies, although survival bias can still be a problem.^41^ Julian *et al*.^27^ discovered that high-intensity, frequent, leisure-time exercise increased an individual’s risk of developing ALS. Other forms of exercise, including occupational PA and movement measured by accelerometer, were not linked to the risk of ALS. The authors had predicted the result based on the observation that the most vulnerable motor neurons in ALS supply fast twitch muscle fibres.^27^ The specificity of this finding helps to counter the possibility that the result was confounded by survival bias.

Julian *et al.*^27^ also applied the HAPAQ in a smaller cohort of *C9orf72*-ALS patients (*n* = 17) where it was demonstrated that age of onset of C9-ALS was inversely correlated with levels of historical PA. This report did not find the same strong relationship in a larger group of sporadic ALS patients suggesting that there may be a specific interaction between the *C9orf72* genotype and PA. These findings need to be validated in a larger cohort of *C9orf72*-ALS patients.

A large-scale cohort study from Mattsson *et al*.^15^ assessed whether PA was implicated in the aetiology of ALS. The compulsory Swedish military service conscription physical exam, taken at the age of 18, was used for recruitment (*n* = 684 459). This increases the power of their study and reduces selection bias. The measurement of physical fitness used was the maximum load that conscripts could sustain for 6 min. This has good reliability and correlates well with other endurance tests.^42^ They concluded that weight-adjusted physical fitness (OR 1.81, 95% CI 1.07–3.06) was a risk factor for ALS, but not PA *per se*. Despite the large sample size, PA was not assessed after the age of 18 years of age, hence the quantity or intensity of PA carried out in these individuals was not assessed longitudinally. The data were collected from men born between 1951 and 1965 and followed up until 31 December 2006, which would allow the oldest participant to be 55 years. Eighty-five males were identified as dying from ALS in this period. However, as ALS commonly affects individuals ∼60–75 years of age, a longer follow-up period would be needed to ascertain the true risk.^12,13^ The study design does not account for potential confounding variables, for example smoking or genetic factors. The authors do however highlight the possibility of an ‘athletic phenotype,’ implying that a particular genetic profile may contribute to sporting ability and the propensity to develop ALS.^15^

A cross-sectional study conducted by Raymond *et al*.^24^ focused specifically on vigorous PA in ALS participants. They used the National ALS registry, allowing for a large sample size, with 8739 participants completing at least one survey. They found a statistically significant difference between vigorous PA in the years 15–24 and 25–34 and diagnosis of earlier-onset ALS (*P* = 0.0009 and *P* = 0.0144, respectively). They utilized a global PA questionnaire designed, validated, and evaluated by the World Health Organization. The lack of a validated questionnaire is commonly a limitation in many of the studies relating to ALS and exercise.

A cohort study was conducted by Turner *et al*.^34^ to assess whether ALS symptoms occurred in the dominant upper-limb or foot. In upper-limb onset patients they found a concordance for side of onset and dominant hand (97/151 or 64%, *P* ≤ 0.0006). However, in lower limb-onset patients this was not present (99/181 or 55% *P* = 0.234). They concluded that PA is linked to ALS, as physical demands on upper limbs are heavily influenced by limb dominance, whereas the commonest physical demand on lower limbs is standing, therefore this demand is distributed more equally between the right and left limbs. This study involved 502 participants and had a high response rate. The approach was prone to recall bias, as it is likely the symptoms would be noticed sooner in the dominant hand, due to greater use and awareness. The authors blinded the participants to the hypothesis, to reduce this potentially confounding element.

A recent prospective case-control study assessed the causality of lifestyle factors in the pre-symptomatic phase of ALS, in participants with the *C9orf72* mutation.^28^ They compared C9-positive ALS patients (*n* = 143), C9-negative ALS patients (*n* = 1322) and healthy controls (*n* = 1322). Participants were assessed for 50 years pre-onset. The authors found that daily energy expenditure at symptom onset was significantly higher in the C9-positive (712 kJ, 95% CI 212–1213, *P* = 0.0053) and C9-negative ALS groups (497 kJ, CI 295–700, *P* < 0.0001) in comparison to healthy controls. The C9-positive group was a much smaller cohort, and therefore potentially this study was underpowered. This study highlights that, in this Dutch population, increased levels of physical exercise and having a *C9orf72* mutation increased the likelihood of developing ALS. This paper only focused on the *C9orf72* genetic subtype, and other genetic causes of ALS and polygenic risk factors were not considered.

Many sports are not discussed in this review as they have not been researched extensively. It would be interesting to investigate whether resistance training such as used by body builders is a risk factor for ALS. Julian *et al*.^27^compared infrequent ‘strenuous sport’ or heavy DIY and frequent leisure-time PA. They found that infrequent ‘strenuous sport’ or heavy DIY were not significantly associated with ALS, but frequent leisure-time PA was linked.^27^ These forms of more static exercise would be most similar to activities such as resistance training. This is an area that requires further research.

Overall, the evidence from the studies described above suggests that there is a positive correlation between total, strenuous PA over the life course and ALS risk, when a risk-genotype is present. Negative reported findings are likely due to underpowered studies, with unvalidated measures of PA, that have not focused on specific genotypes.^16–18,35^

### Soccer and American football

Many professional athletes have developed ALS, with multiple reports specifically focusing on soccer players. Therefore, soccer specifically, rather than overall PA, has been suggested as a risk factor for the development of ALS. Soccer is predominantly an aerobic form of exercise, although anaerobic exercise occurs when players are performing sprints.^43^ There has been speculation about whether it is the type of PA undertaken by soccer players that increases the risk of ALS, or whether the association could be due to their increased risk of head injuries caused by heading the ball, the use of illicit performance-enhancing drugs or players continuous contact with grass (with potential increased exposure to pesticides and toxins used to maintain the grass).^30,44–46^

A recent case-control study conducted by Filippini *et al*.^47^ assessed non-genetic ALS risk factors, in 95 ALS cases and 135 controls. They found a slight increased risk of ALS associated with soccer at a competitive level (OR = 1.19, 95% CI 0.35–4.02), but an inverse association with sport in general (OR = 0.77, 95% CI 0.41–1.42). This implies that the specific type of exercise involved in competitive soccer carries a higher risk for the development of ALS. This study is limited as it does not explain how the exercise exposure data were collected and included only a small sample size of 95 ALS cases due to a low response rate (18.9%). The responses were also self-reported, allowing for recall bias. There was no information provided regarding position in the football team (which can be used to infer whether it was due to higher anaerobic or aerobic activity), risk of head injury or the time between playing soccer and diagnosis; this interval may not have been long enough.

A retrospective cohort study conducted by Daneshvar *et al.*^23^ focused on NFL athletes, looking at incidence and mortality from ALS. They had large sample of 19 423 male former NFL athletes. They found that the incidence was four times as high as that of the general US population (standardized incidence ratio, 3.59, 95% CI, 2.58–4.93). Those diagnosed with ALS were also found to have a longer career (average of 7 years versus 4.5 years for those who did not develop ALS). Any other PA was not accounted for outside of NFL playing and the exercise itself was not quantified. Despite this, the large sample and positive findings imply that the association between NFL and ALS is most likely related to the large quantity of strenuous exercise performed over several years.

A prospective cohort study by Chio *et al*.^14^ assessed the risk of ALS in Italian soccer players (*n* = 7325), in comparison to road cyclists (*n* = 1701) and basketball players (*n* = 1973). There was a significantly increased risk of developing ALS at a younger age in soccer players, with a predilection for mid-field players. This risk was not seen in road cyclists or basketball players. Therefore, the type, frequency and mechanisms of PA appear important in relation to ALS risk. Basketball is mostly an anaerobic exercise, characterized by a lower maximum rate of oxygen consumption (VO_2MAX_), whereas road cyclists have a higher VO_2MAX_ compared to soccer players. Previous studies have highlighted that midfielder soccer players require more aerobic activity and that they have a low anaerobic capacity.^48^ Therefore, it may specifically be intense aerobic activity that is a risk factor for ALS. This study does not explain how the amount of PA was quantified, or whether this was considered. Therefore, the soccer players may have had a higher frequency of strenuous exercise, over a longer time period. This study did not include genetic profiling, despite the *C9orf72* genetic mutations having a relatively high prevalence in Italy compared to other countries.^49^ Another study focusing on soccer players, found that neurodegenerative disease was highest for defenders [hazard ratio (HR) 4.98 95% CI, 3.18–7.79, *P* < 0.001] and lowest for goalkeepers (HR 1.83, 95% CI 0.93–3.6, *P* = 0.08) implying that anaerobic activity is more important in the causation of ALS.^26^ This study had a much larger sample size of 30 704 males, therefore the results have higher statistical power. The authors also reported that the risk of ALS was higher, when soccer players have professional careers lasting over 15 years (HR 5.20, 95% CI, 3.17–8.51, *P* < 0.001).^26^

Further reports strengthen this link between soccer and ALS. A recent prospective cohort study found that the mean age of onset of ALS in soccer players was 23.7 years younger compared to cases within the general European population (*P* < 0.0001).^25^ A case report described three amateur league soccer players from the same team in Southern England who all developed ALS.^45^ Another report focusing on mortality from neurodegenerative diseases of retired American football players, found that ALS was four times more common compared to the general US population.^33^ The specific cause of this link is not yet defined.

Overall, these studies support the hypothesis that soccer and American football are linked to the development of ALS more than other forms of exercise. This does not rule out a role for PA more broadly, but suggests that soccer may have a larger effect-size. In light of this, we suggest that efforts to profile at-risk individuals should focus on soccer players specifically, given the global popularity of this sporting activity. The effects of aerobic versus anaerobic exercise need to be further investigated. There is conflicting evidence regarding the effect of the team position, although differences in sample sizes make direct comparisons difficult.

### Skiing

Fang *et al.*^30^ conducted a large scale (*n* = 508 239) case-control study, assessing whether cross-country skiers had an increased risk of developing ALS in Sweden. Cross-country skiing involves a combination of sustained aerobic energy turnover and repeated work rates above the VO_2peak_ (anaerobic activity). This differentiates cross-country skiing from other endurance sports.^50^ This type of PA burns the most calories per hour in comparison to other competitive sports.^30^ Both head trauma and use of illicit performance enhancing drugs are uncommon in skiing compared to soccer.^30^ This study was conducted to investigate whether it was soccer and head injuries specifically that increased the risk of ALS or whether it is the type of PA undertaken. Fang *et al*.^30^ found that faster skiers had a 4-fold increased risk of developing ALS (HR 4.31, 95% CI 1.78–10.4). This supports the concept that strenuous PA is a risk factor for ALS in line with the work of Julian *et al*.^27^ previously mentioned. The analysis also adjusted for confounding variables, including education, employment, and region of residence. Additionally, the skiers who participated in more than four races (HR 3.13, 95% CI 1.37–7.17) in comparison to one race had a higher risk of developing ALS.

In preclinical studies moderate exercise has been shown to protect the neuromuscular system, whereas endurance exercise has been shown to promote oxidative stress and enhance motor neuron degeneration.^51^ This offers an explanation for the increased risk of ALS described in the report from Fang and colleagues and strengthens the argument that strenuous, frequent PA enhances ALS risk, regardless of whether the specific type of PA predisposes to head injury. Moreover, the suggestion of a dose effect is good evidence which is consistent with the study described above linking the length of a soccer career to ALS risk.

### Chronic traumatic encephalopathy: trauma rather than the exercise?

Several studies have assessed the relationship between the exposure to repetitive head injury, rather than PA itself, as a risk factor for ALS. Chronic traumatic encephalopathy (CTE) specifically is a form of neurodegeneration triggered by repetitive head injury. A 12-person case study of CTE showed that 10 of these cases had widespread TAR DNA-binding protein 43 (TDP-43) pathology, with three of these individuals developing progressive ALS several years before death.^52^ Nuclear depletion and cytoplasmic aggregation of TDP-43 within motor neurons represent the hallmark pathology of ALS.^53^ However, it should be pointed out that the population of neurons affected by CTE is distinct from ALS. The evidence to date does not indicate that CTE is a cause for ALS, but rather that both conditions cause the development of TDP-43 proteinopathy.

Multiple studies focusing on retired football players, found that players who had reported at least three episodes of concussion were significantly more likely to report cognitive symptoms in later life.^54–56^ These symptoms could be related to CTE or ALS. Savica *et al*.^16^ conducted a cohort study focusing on whether the incidence of ALS was higher in high school American football players in the years 1946–56, before protective headgear was used. They found no increased risk of ALS in comparison to the control population. The severity of any concussion injuries was not detailed. This report also stated that the controls were ‘non-football’ playing, but it does not describe their PA levels. However, this study was focusing on head injury and not PA, and the report concludes that increased head injuries during football does not increase the risk of ALS.

Lehman *et al*.^33^ analysed the neurodegenerative causes of death among a cohort of professional American football players. They found a 4-fold increase in mortality from ALS in comparison to the general US population. They hypothesized that head injury was a risk factor and found that there was a higher mortality from neurodegenerative disease in those who had held speed positions compared to those with non-speed positions. They suggested that speed positions have a higher risk of concussion injuries, though this was not specifically evaluated. They did not document the numbers of head injuries or episodes of concussion. It should be noted that speed positions also require more strenuous, anaerobic activity which may relate to the increased risk of ALS.

The study of Julian *et al.*^27^ used MR to link ALS to frequent strenuous PA. By definition, MR implicates a linear dose-response relationship, whereas the activities included in this exposure were diverse and will have had wide variance in their association with head injury. Therefore, head injury could not have caused this dose-response relationship, as it is very unlikely that this would occur as a linear function of PA.

Filippini *et al*.^47^ conducted a case-control study in Italy. They found a positive association between ALS risk and head trauma (OR = 2.61, 95% CI 1.19–5.72). This study did not give methodological details of the instrument used to assess exposure to head trauma. It was also self-reported, allowing for recall bias. The sample size was small (*n* = 95) with a low response rate, which impacts upon the precision of the risk estimates. Another study focusing on American high-school football players, found that they had an increased risk of medically documented trauma, but not an increased risk of neurodegenerative disease.^18^ Therefore, trauma or head injury itself was considered unlikely to be a risk factor for ALS. The controls were other male athletes at high school, for example swimmers and basketball players. Therefore, this study does not address whether PA is a factor for the risk of developing ALS.

To summarize, the evidence to date suggests that it is most likely the type of PA involved in the sports which can be associated with head injuries, that increases the risk of ALS, not the head injuries themselves. These studies highlight common problems with epidemiological studies in ALS. Conflicting results often arise because of low powered studies.

### Normal physiological responses to exercise

**Figure 2:**
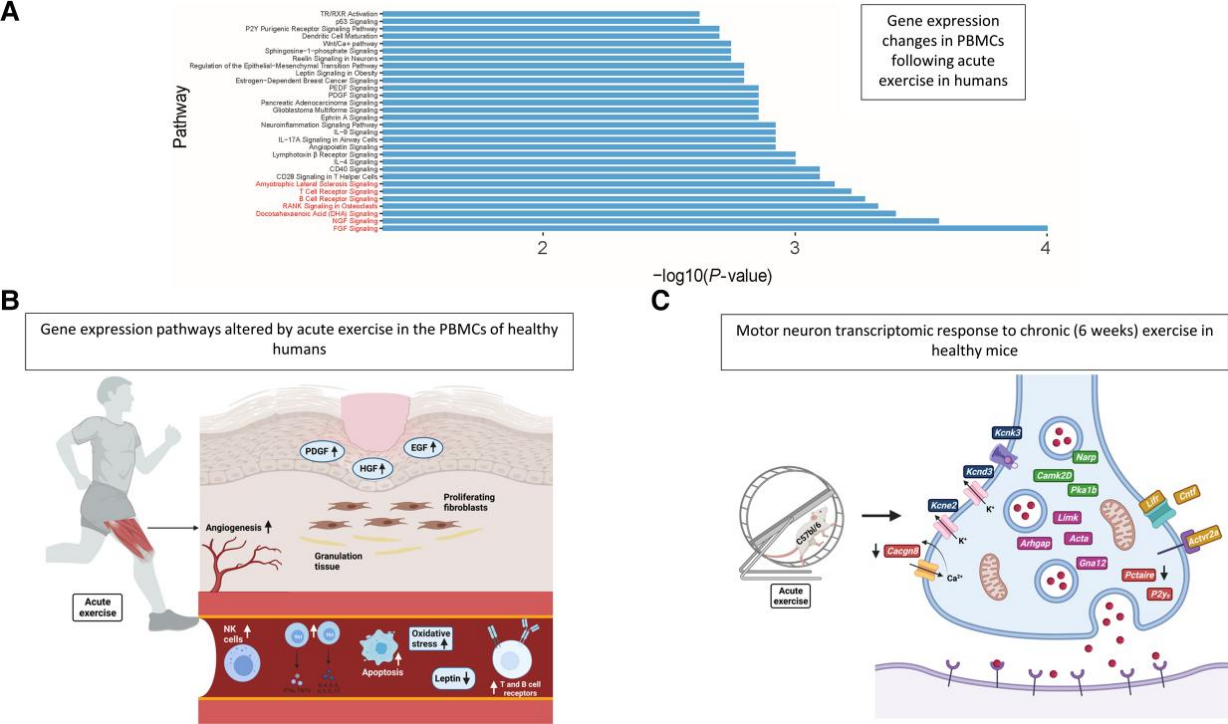
**Physiological response to acute exercise in healthy humans and mice.** (**A**) Transcriptome analysis of peripheral blood mononuclear cells (PMBCs)^57^ reveals that 22% of the biological pathways differentially expressed following acute exercise are significantly enriched with ALS-associated rare genetic variants. The top 30 pathways by *P*-value are shown. All pathways depicted pass multiple testing correction (FDR < 0.05). (**B**) The physiological impact of acute exercise in humans measured by gene expression changes in PBMCs. Acute exercise induces an inflammatory response [increasing natural killer (NK) cells, Th1 and Th2 activation, B cell receptors, T cell receptors]. It also increases oxidative stress, apoptosis and upregulates angiogenesis and wound healing pathways [platelet-derived growth factor (PDGF), hepatocyte growth factor (HGF) and epidermal growth factor (EGF)] signalling and dysregulates metabolic pathways, decreasing leptin.^57^ (**C**) The important gene expression changes in the motor neurons from mice following prolonged regular daily exercise for 6 weeks. Mouse data are depicted as it is not possible to obtain details of the motor neuron transcriptome from living human subjects. The neurotrophic factors and receptors (Cntf, Lifr, Actvr2a) are increased. Genes involved in signalling (Pka1b, Narp, CamkIId) are also increased. Genes involved in ion channels (Kcnd3, Kcne2, Kcnk3) are increased and (Cacng8) is decreased. Genes involved in cytoskeleton reorganization (Arhgap, Gna12, Limk, Acta) are increased, these are involved in neurite outgrowth. P2Y9 and Pctaire, involved in neurite retraction, are decreased.^58^

PA is a key factor in maintaining health across the lifespan. Regular moderate-intensity training reduces oxidative stress, decreases inflammatory markers such as interleukin-6, and helps to preserve cardiovascular fitness and brain function.^51^ When undergoing PA, the neuromuscular system experiences significant structural and functional alterations (Fig. 2). These responses optimize the transmission of electrical and biochemical stimuli and promote axonal outgrowth.^58^ The intensity and type of PA lead to different alterations in the transcriptome.^58^ It has been hypothesized that the normal physiological stress response to PA is impaired in individuals predisposed to develop ALS, which could explain a proposed gene-environment interaction leading to exercise-induced ALS.^59^ Indeed, Julian and colleagues previously demonstrated that genes, which are differentially expressed in response to exercise, are enriched with rare deleterious mutations in ALS patients.^27^ This observation enabled identification of a series of biological pathways underpinning this interaction; the most significant changes were within nerve growth factor (NGF) and fibroblast growth factor (FGF) signalling pathways, which have been implicated in chronic adaptation to repeated exercise.^60,61^ The biological pathways implicated in this study are shown in Fig. 2A (adapted from Julian *et al*.^27^).

It is difficult to study gene expression changes in response to PA in human muscle and motor neurons. *In vivo* studies in mice found that motor neurons respond to PA by synaptic reorganization, electrophysical changes and changes in neurotrophic signalling. These changes differed between acute and chronic exercise.^58,62^ Microarray technology and laser capture microdissection were combined to analyse response of motor neurons and skeletal muscle in 12 10-week-old (C57bl/6 strain) mice. Following a 6-week period of daily exercise mice were shown to upregulate 203 genes and downregulate 241 genes within motor neurons. The differentially expressed genes were mainly involved in signalling, the cytoskeleton, transcriptional regulation and synapse reorganization. The neuromuscular junction (NMJ) represents a specialized type of synapse. In mice following exercise a significant number of genes involving NMJ functioning, and development are transcriptionally altered.^58^ In chronic exercise for example, the catenin-cadherin system is activated. This is involved in cell-to-cell contact, neurite outgrowth and NMJ consolidation. Gene expression changes were also seen in the skeletal muscle following exercise. The gastrocnemius muscle showed upregulation of several protocadherins (Pcdh1, 12, 17, 18), which are involved in synapse formation. Therefore, chronic exercise alters synaptic reorganization and transcriptional regulation. Other important genes that are upregulated in muscle during exercise are *Cntf* and *Lifr*. These aid in the response to the physiological stress of intense exercise and mutations in these genes have been shown to be disease modifiers in ALS, leading to an increased risk and earlier age of onset.^58^

A recent report investigated the molecular choreography of acute exercise with longitudinal multi-omic profiling of plasma and PBMCs in 36 participants before and after controlled PA.^57^ The authors highlighted that exercise induced a robust inflammatory response (increasing natural killer cells, Th1 and Th2 activation, B cell receptors, T cell receptors, nuclear factor kappa-b signalling and interleukin signalling pathways). PA also triggered early release into the circulation of pro- and anti-inflammatory proteins, increasing oxidative stress and propensity to apoptosis. They found that these gene expression changes were present acutely post-exercise, and the majority returned to baseline within 30 min post-exercise.^57^

### Potential pathophysiological responses to physical activity in ALS

#### Biological pathway changes

Different types of exercise can impact various biological pathways and different subtypes of motor neurons. FGF and NGF pathways are enriched in PBMCs following acute exercise and have been shown *in vitro* astrocytes to cause motor neuron apoptosis, which has previously been implicated in the pathophysiology of ALS (Fig. 2).^27,63,64^ FGF1 secretion is stimulated by oxidative stress, hypoxia and serum starvation.

**Figure 3:**
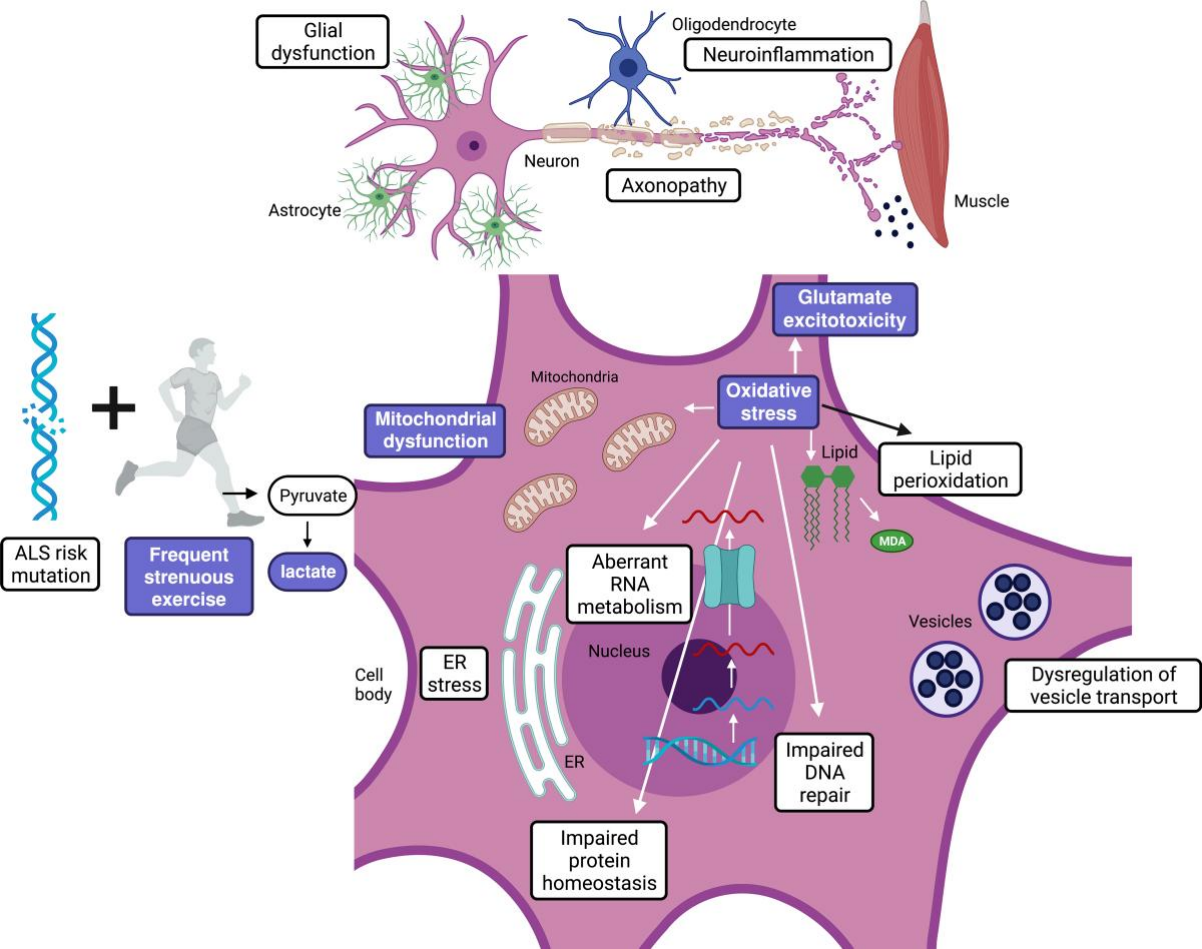
**The pathophysiological mechanism of ALS.** The complex pathophysiological mechanisms in ALS. The labels highlighted in bold show which pathological mechanisms in ALS are increased further by strenuous exercise. ER = endoplasmic reticulum.

Matrix reorganization and the response to hypoxia are the main identified pathways activated in response to PA in healthy individuals.^65^ Mutations or other perturbations in these pathways in ALS, could lead to differential expression in response to hypoxia, oxidative stress and depletion of nutrients/cellular starvation during exercise, precipitating motor neuron injury (Fig. 3).^20^

NMJ remodelling is an adaptive response to exercise. In previous studies in a pathological (G_4_C_2_)_48_*Drosophila* model and (G_4_C_2_)_48_-MS2 rat spinal cord, G_4_C_2_ repeat expansions of *C9ORF72* were shown to cause deficits in dendritic branching and therefore defective remodelling of the NMJ and synaptic dysfunction.^66^ This defective remodelling in patients with genetic mutations, such as those found in *C9orf72*, is likely to become a pathological phenotype when exposure occurs to the stress caused by exercise.^58^ In animal models of ALS, there is evidence of NMJ denervation and dysregulation of proteins involved in synaptic maintenance and plasticity.^67,68^ These studies highlight that, in the presence of genetic changes causative for ALS, the neuromuscular system may be unable to respond to the stress produced by PA in the same way as a healthy individual. This may explain the genetic–environmental interaction predisposing to the development of ALS.

#### Skeletal muscle changes

Muscle fibres and motor neurons are sub-specialized for aerobic and anaerobic conditions. Skeletal muscle fibres are classified as either fast twitch (type IIa, IIb and IIx) or slow twitch (type I) according to their contractile speed and metabolic properties.^69^ In the early disease process, ALS is known to selectively affect the motor neurons responsible for supplying fast twitch muscle fibres (type IIb).^70,71^ These are used for anaerobic burst activity, high intensity exercise.^70,71^ Julian *et al*.^27^ in a recent MR study found that high-intensity, high-frequency, leisure-time exercise led to an increased risk of developing ALS. This type of activity involves the use of type IIb muscle fibres.^58,62^ Therefore, acute intense anaerobic exercise specifically stresses the motor neuron group most vulnerable to the disease process in ALS.

#### Dysregulation of energy metabolism

A study in the SOD1^G93A^ mouse model of ALS showed an increase in mitochondrial mass and of mitochondrial DNA in different muscles during the course of the disease.^72^ Nicotinamide adenine dinucleotide dehydrogenase-tetrazolium reductase (NADH-TR) reflects the concentration of mitochondria within myocytes and succinate dehydrogenase (SDH) is a key enzyme in the Krebs cycle. The authors found that the activities of these enzymes in skeletal muscles were dramatically increased in the early pre-symptomatic stage of ALS (55 days).^72^ This hypermetabolism and its links to PA can also be seen in a case-control study evaluating the metabolic correlates of lifetime sport in ALS through brain ^18^F-FDG-PET scans.^29^ There was significant cerebellar hypermetabolism in the group who did not exercise compared to the group who did. This hypermetabolism correlated with more significant and widespread metabolic changes in areas known to be affected by ALS, the frontotemporal regions and corticospinal tracts. Despite this hypermetabolism, both groups expressed the same level of disability. The authors therefore concluded that participants who did not exercise coped better with the neurodegenerative process in ALS, inferring that PA has an additive effect to underlying changes in metabolism.

Professional athletes are known to be exposed to high levels of metabolic stress, and as described above, ALS appears to be more common within these individuals. Patients with ALS mutations have defects in energy metabolism, and an inability to respond to the additional metabolic stress of PA may be the cause of the propensity of these athletes to develop ALS at a younger age.^73,74^ Morphological changes consistent with increased protein synthesis during exercise have been seen in the motor neurons of treadmill running rats.^75^ SDH was also increased in exercised skeletal muscle to compensate for the increased energy demand. If repetitive acute exercise is conducted (regular motor neuronal excitability), it may cause the motor neurons to be less excitable when at rest. Therefore, the effects continue when PA is stopped. These *in vivo* studies strengthen the argument that intense, strenuous PA combined with an underlying genetic mutation for ALS, can increase the risk of developing the disease.

An *in vivo* study of SOD1_G93A_ transgenic mice showed that running-based training increased their survival by delaying the onset of the disease. However, high endurance exercise had a detrimental effect on survival rate.^76,77^ These data suggest that high intensity exercise specifically may be detrimental in the presence of a SOD1 mutation—though this has not yet been explored in humans. Another study conducted in SOD1_G93A_ transgenic mice showed a massive upregulation of energy generating pathways in motor neurons at the early pre-symptomatic stage,^78^ increasing our understanding of the impact that strenuous PA may have in an already stressed metabolic state.

Animal studies, for example in SOD1 mice exposed to exercise, have not consistently shown a detrimental effect of exercise on the disease phenotype. One study compared the effects of swimming-based training and moderate running-based training in the SOD1 ALS mouse model. They found that swimming significantly delayed motor neuron death, maintaining the fast phenotype in fast-twitch muscles and increased life span by ∼25 days.^79^ These findings may be due to the mutations affecting the slow-twitch muscles fibres primarily and therefore only being affected by specific sporting activities that trigger these specific fibre types. Overall, these studies highlight that specific high endurance PA and an ALS mutation, can potentially cause a toxic increase in energy generating pathways.

#### Oxidative stress

If a person with an ALS risk-genotype undertakes vigorous exercise, this predisposes to hypoxia, cellular energy starvation and an increase in oxidative stress. An inability to respond appropriately to these stresses may predispose to motor neuron injury.^27^ Abnormal biomarkers of oxidative injury within CNS tissues in ALS, have been shown in both post-mortem and *in vivo* studies.^20^ The elevated oxygen consumption and tissue metabolism required to perform vigorous exercise augments the generation of reactive oxygen species (ROS). Acute exercise is known to induce a transient increase in ROS, and individuals with chronic disease experience an exacerbation in oxidative stress following acute exercise in comparison to healthy individuals.^32^ During vigorous PA the normal adaptive processes seen in regular moderate exercise may not be achieved, causing deleterious effects of ROS.^20^ This excess of ROS can cause DNA and RNA modification, lipid peroxidation and oxidation and nitration of amino acids.^80^ This causes an accumulation of products of oxidative damage, causing further mitochondrial dysfunction and contributing to neurodegeneration (Fig. 3).^81,82^

Pasquinelli *et al*.^32^ conducted a study looking at markers of oxidative stress in plasma and how they differed in exercise fatigue testing in ALS patients. They found that in ALS patients there were significantly increased advanced oxidation protein products (*P* < 0.001), decreased ferric reducing ability (*P* < 0.001) and thiol groups (*P* < 0.001) in comparison to controls. This study indicates a decrease in antioxidant defences in ALS patients. The peroxisome proliferator-activated y co-activator 1α (PCG-1α) was specifically investigated, as it has previously been implicated in the pathogenesis of ALS.^83^ PGC-1α protects the mitochondria from oxidative stress by reducing the accumulation of ROS and apoptotic cell death.^83^ During inactivity PGC-1α is located in the cytoplasm, and it is activated during muscle contraction and relocates to the nucleus where it promotes the transcription of genes involved in skeletal muscle fibre remodelling, antioxidant defence, mitochondrial biogenesis and oxidative phosphorylation.^32^ The authors assessed different single nucleotide polymorphisms (SNPs) of PCG-1α. They found that lactate levels were significantly higher (*P* < 0.01 and *P* < 0.001) and greater protein oxidative products were found in patients with the AA (Ser482Ser) allele of PCG-1α compared to individuals with the GG (Gly482Gly) and GA (Gly482Ser) genotypes, which is consistent with greater exercise-related oxidative stress.^32^ In previous studies, the SNP change from G > A has been shown to inhibit the transcription of antioxidant genes and subsequently increase oxidative stress.^84^ Therefore specific mutations causing an increase in oxidative stress were found in ALS patients in comparison to controls.

It has been proposed that vigorous PA might exacerbate glutamate excitotoxicity, which is known to contribute to motor neuron injury in ALS.^85^ Overstimulation of glutamate receptors promotes an abnormal calcium ion influx into neurons, with adverse effects on mitochondrial function, increased oxidative stress and ultimately neuronal cell death.^20^ Motor neurons are particularly susceptible to excitotoxicity, given their high expression of calcium-permeable AMPA receptors lacking the GluR2 subunit and their relatively low expression of calcium buffering proteins.^86^ Overall, vigorous PA likely increases glutamate excitotoxicity and oxidative stress, hastening neurodegeneration in individuals with a genetic predisposition for the development of ALS.

### Specific ALS genetic subtypes and mutations and physical activity

ALS is classified into sporadic (90–95%) and familial (FALS, 5–10%).^5^ At least 30 genes have been found to cause FALS, of which the most common is the intronic hexanucleotide expansion within the *C9orf72* gene which is present in ∼40% of FALS cases and ∼7% of ALS cases with apparently sporadic disease. The penetrance of disease in the presence of *C9orf72* mutations is not complete, although ∼99.5% of carriers will manifest disease by the age of 83 years.^87^ One study reported that 0.6% of healthy individuals in the UK carry an abnormal *C9orf72* expansion.^10^ Most laboratories consider a GGGGCC repeat length of <30 in blood to be normal, with >30 repeats likely to be pathogenic.^88^ It is likely therefore that additional environmental factors may affect the penetrance of disease.^6,12,89,90^

Julian *et al*.^27^ focused on enrichment of ALS genetic risk (specifically *C9orf72*) with exercise-associated transcriptome changes. They highlighted the report of gene expression changes of known ALS genes post-exercise in PBMCs from normal individuals where *C9orf72* was downregulated significantly following acute exercise (fold change = −0.2, false discovery rate = 0.0002).^57^ They also reported that, when using the validated HAPAQ questionnaire, the age of onset was inversely proportional to levels of historical PA for *C9orf72*-ALS (Cox proportional hazards model, Wald test *P* = 0.007, likelihood ratio test *P* = 0.01, concordance = 74%) but not for non-*C9orf72*-ALS.

A case-control study assessed the effect of lifestyle factors on causation stratified by the *C9orf72* mutation.^28^ The authors found that daily energy intake at symptom onset was higher in the C9-positive (712 kJ, 95% CI 212–1213, *P* = 0.0053) and C9-negative groups (497 kJ, CI 295–700, *P* < 0.0001), concluding that a combination of increased PA and an underlying *C9orf72* mutation can increase the risk of developing ALS. There were only 143 patients in the *C9orf72* group, decreasing the power of the study. These studies only focused on one genetic cause of ALS and the interaction of PA with other genetic causes of ALS needs to be evaluated in the future.

TDP-43 is the hallmark pathology of ALS.^53^ Two very recent studies have linked nuclear loss of TDP-43 to key splicing changes, leading to loss of function of genes including *STMN2* and *UNC13A*, with consequences for clinical severity.^91,92^ TDP-43 is mislocalized to the cytoplasm, where it exhibits a toxic gain-of-function, contributing to neuronal injury and cell death.^93,94^ TDP-43 mislocalization is usually reversible in most model systems, however in both CTE and ALS, this mislocalization forms into potentially permanent pathological cytoplasmic aggregates.^93^ Importantly the quantification of these aggregates predicts the degree of neuronal loss.^95^ The specific effects that exercise has on TDP-43 aggregates is not currently known. The ability to specifically define the gene–environment interaction, involving exercise, that leads to this potentially irreversible TDP-43 mislocalization could allow a deeper understanding of the pathophysiology of ALS and aid with treatment discovery. One study previously attempted this; however, brain and spinal cord tissue was only obtained from 14 ALS patients.^96^

## Conclusions and future directions

The only epidemiological risk factors currently established for ALS are increasing age and male sex. The ability to establish further risk factors, would allow for more personalized medicine and potentially preventative strategies for this devastating disease.

Key points emerging from this review include: (i) the balance of evidence over the past 13 years favours the conclusion that participation in frequent and strenuous leisure-time PA is a risk factor for the development of ALS; (ii) specific genetic backgrounds predisposing to exercise-induced ALS are being identified with early data highlighting the interaction with *C9orf72* mutations; (iii) specific strenuous PAs that have been studied in the context of ALS include soccer, American football and long-distance skiing. Other sports with different anaerobic versus aerobic components should be studied in future; and (iv) the risk for ALS is not confined to impact sports with a high propensity for head injury.

Current evidence for PA as a risk factor for ALS has increased in depth and quantity over the last few years. This review highlights the common problems faced in researching this topic, for example the relatively low prevalence of ALS, the challenges of measuring PA over the life-course, with unvalidated questionnaires and recall bias likely impacting the results obtained. The validated HAPAQ questionnaire has been used in a few of these studies and will allow historical PA levels to be measured with a reasonable degree of accuracy. Despite the issues the studies face, it is clear from the balance of all the evidence, that PA does play a part in the pathogenesis of ALS, when the participant has an underlying at-risk phenotype.

**Figure 4:**
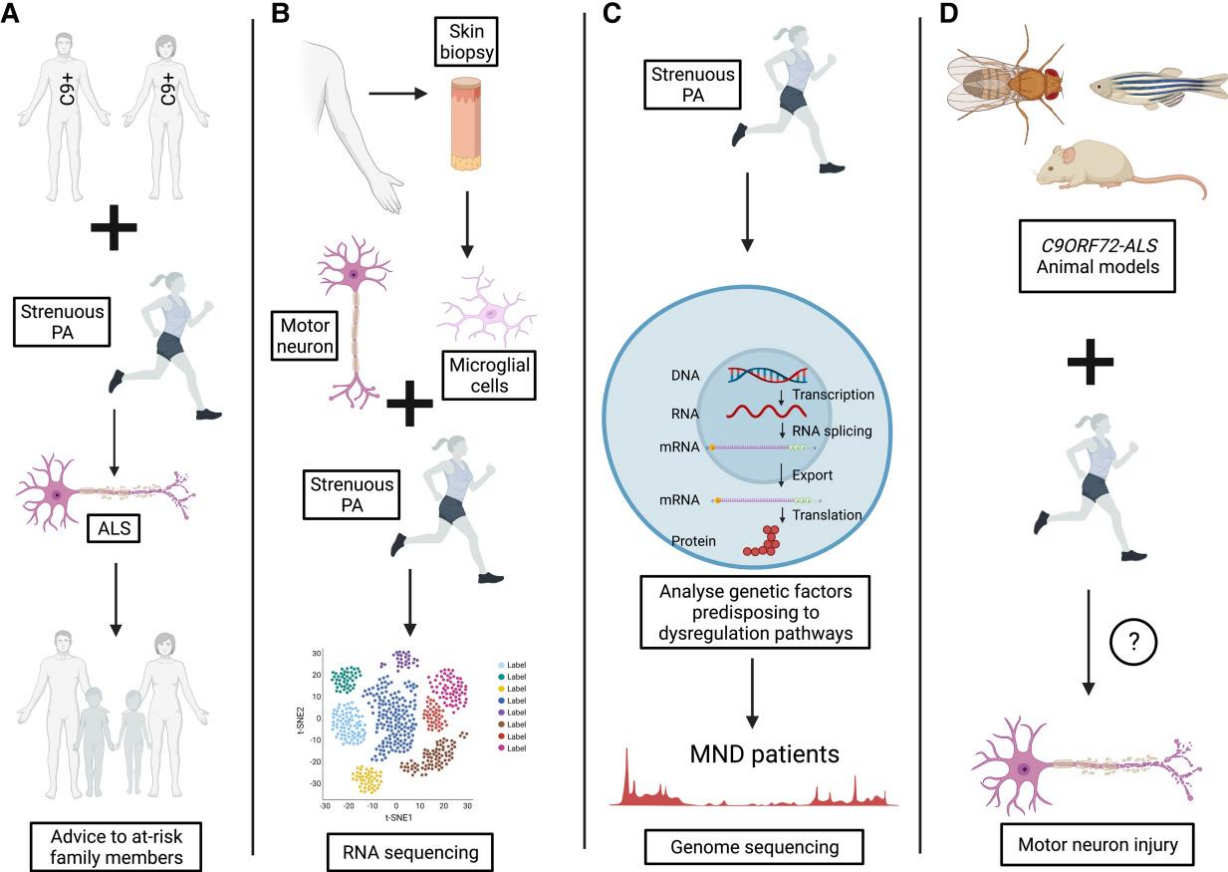
**Future recommendations.** (**A**) A large sample of C9ORF72-ALS patients should be studied using a validated questionnaire, such as the HAPAQ, to establish in a larger scale study whether strenuous PA exacerbates the clinical phenotype. This may allow lifestyle advice to be provided to at-risk family members with this genetic subtype of ALS. (**B**) Skin biopsies can be taken from participants with ALS and healthy controls. The fibroblasts can be reprogrammed to generate motor neurons, astrocytes and microglial cells. These human cell models can be studied to assess in detail pathophysiological changes when subjected to stresses e.g. hypoxia and oxidative stress, which operate during strenuous PA. The differences in stress response can be analysing using RNA sequencing, investigating any dysregulation of the response transcriptome in cell models of ALS compared to controls. (**C**) There are known transcriptomic changes present in PBMCs following strenuous exercise. The genetic factors predisposing to dysregulation of the physiological pathways can be analysed in large datasets of whole genome sequencing from a large sample of ALS patients. (**D**) Animal models of genetic subtypes of ALS beyond SOD1 can be studied to evaluate the effect of strenuous PA on disease parameters such as age of onset and survival. For example, for *C9ORF72* ALS relevant *Drosophila*, zebrafish and mouse models have been generated, which will allow evaluation of this genetic–environmental interaction.^97–100^ Figure created using BioRender.com.

Genetic evidence to date on the role of PA as a risk factor for ALS, has focused on the most common genetic cause of ALS—mutations of the *C9orf72* gene. However, the majority of ALS has a polygenic architecture^96^ and future work will consider the interaction of exercise with the full spectrum of genetic changes linked to ALS (Fig. 4). The advent of large population-scale biobanks including whole genome sequencing such as UK Biobank (UKB), is a significant step towards this aim because of the size which facilitates prospective analysis, and the systematic capture of environmental exposure information, including exercise. Currently, with median follow-up of 11.9 years, 343 of the UKB participants have developed ALS following enrolment.

It is likely that specific forms of exercise are important, for example anaerobic reactions in strenuous activity, and factors such as sports-related head injury are not. In the future, large scale studies focusing on specific at-risk genotypes and PA should be conducted to confirm this association (Fig. 4). This will strengthen the evidence currently available and allow advice to be given to the at-risk family members of the patients with these subtypes of ALS. Increasing our understanding of the pathophysiology of ALS and potential genetic-environmental interactions will allow for the possibility of developing preventative therapeutic approaches in the future.

## Supplementary Material

awac470_Supplementary_Data
