# Supplementary material for: Physical activity as an exogenous risk factor for amyotrophic lateral sclerosis: a review of the evidence
Source: Brain. 2023 Feb 22;146(5):1745–57. doi: 10.1093/brain/awac470 (PMC10151186; doi:10.1093/brain/awac470)
Supplement: awac470_Supplementary_Data [file awac470_supplementary_data.pdf]

Supplementary material

Supplementary Table 1*: Summary of the papers reviewed, assessing the link between physical activity as a risk factor for amyotrophic lateral sclerosis.* *The studies are listed in descending order from most recent.*

Author	Study design	Aims	Findings	Strengths	Weaknesses
Daneshvar et al., 2021[42]	Cohort study and nested case-control study	To assess the incidence and mortality of ALS in National Football League (NFL) athletes.	• There was a significantly higher incidence (standardised incidence ratio, 3.59; 95% CI, 2.58-4.93) and mortality (standardised mortality ration, 3.94; 95% CI, 2.62-5.69) associated with ALS among NFL players compared to the general US population.<br>• The participants who developed ALS had significantly longer careers (average of 7.0 years) than those without ALS (4.5 years, odds ratio 1.2, 95% CI 1.1-1.3).<br>• No difference in developing ALS for BMI, position played, birth location or race.	• The study included 19,423 athletes.<br>• There was a long follow up period, with average follow up being 30.6 years.<br>• The comprehensive data base Hidden Game Sports/24-7 Baseball LCC was used to access the information.<br>• A further nested case-control study was used to focus on length of career and account for variables, such as BMI and position played.	• The measure of PA included having played 1 or more professional games, this was not specifically quantified and could have varied markedly.<br>• The use of public records to highlight ALS incidence can lead to under-reporting of diagnoses and under-estimate true value.<br>• It is not known how the diagnosis of ALS was made, as no access to clinical documents.<br>• Despite a large sample of athletes, the sample size of ALS players was only 38 cases which is small.
Raymond et al., 2021[32]	Cross-sectional study	To examine the link between early onset ALS and vigorous PA.	• Patients who were involved in vigorous PA at least three times a week were more likely to develop ALS (p<0.0001).<br>• The participants who engaged in at least 3 sessions of vigorous PA per week had a mean age of diagnosis of 56.8 in comparison to less active at 60.5 (p<0.0001).	• The national ALS registry was used allowing a large sample size, with 8,739 participants completing at least one survey.<br>• They used the global PA questionnaire designed by WHO which has been previously validated and evaluated. It covers time periods of PA and duration.	• The surveys were delivered online not by healthcare professionals, potentially increasing recall bias.<br>• The participants therefore needed online access, so the study may be biased towards younger and better educated individuals.<br>• The participants who did not complete the survey were more likely to be overweight or obese, potentially skewing the results.
Gamez et al., 2021[46]	Cohort study	To assess the phenotype of 7 Spanish professional and semi-professional soccer players with known ALS.	• The mean age at onset in soccer players was 23.7 years younger than in the general European population (p <0.0001) and 21.6 years younger than in the Catalan male population (p < 0.0001).<br>• Bulbar onset was less frequent in Spanish league soccer players vs previous studies in Italian players (only present in 1 player).	• Largest sample size of non-Italian professional soccer players.<br>• They considered multiple variables, including family history, age and site of onset, playing position etc.	• Followed up the patients over 20 years, with the average age at the beginning of the study being 18 years old. 38 years is much younger than the age of onset of most ALS patients.<br>• They followed up participants using self-reports and the media, which is prone to recall bias and the media may over or under-exaggerate symptoms.<br>• Very small sample size of only 7 players.<br>• Limited information about PA outside of playing soccer, before or after their career.

Russell et al., 2021[45]	Cohort Study	To assess the neurodegener at-ive disease risk among former professional soccer players.	• 386/7676 former soccer players (5%) and 366/23028 (1·6%) matched controls identified with neurodegenerative disease (HR 3·66, CI 2·88-4·65, p<0·001).<br>• Neurodegenerative disease was highest for defenders (HR 4·98 95% CI, 3·18-7·79, p<0·001).<br>• Neurodegenerative disease was lowest for goalkeepers (HR 1·83, 95% CI 0·93-3·6, p=0·08).<br>• Risk was highest among former soccer players with professional careers >15 years (HR 5·20, 95% CI, 3·17-8·51, p<0·001).	• 30,704 males, large sample size, increasing the power of the study.<br>• Reported their study using the Strengthening the Reporting of Observational Studies in Epidemiology (STROBE) guideline.<br>• All health record data were anonymised to researchers.	• A retrospective cohort study is susceptible to information and recall bias.<br>• Did not have detailed PA history, only dates of signing, retirement and player position.<br>• Did not factor in specific details, for example head injury.<br>• Individuals had to be at >40 years of age, median follow-up time was 18 years, the younger patients may not have developed ALS yet, as average age of onset is 60-75 years.
Julian *et al.*, 2021[26]	Mendelian Randomisation (MR) study	To assess whether strenuous leisure-time activity is a risk factor for the development of ALS.	• G4C2-repeat expansion of *C90RF72* predisposes to exercise-induced ALS.<br>• There is a causal relationship between genetic liability and frequent, strenuous leisure-time exercise causing ALS.<br>• Altered expression in response to acute exercise enriched for ALS risk genes, e.g. *C9ORF72* (p=0·013).<br>• Age of onset inversely proportion to historical PA activity in *C9ORF72*-ALS (p=0·01, concordance=74%).	• Used Mendelian randomisation – evaluating relationship between exposure and outcome and potential causation through upstream genetic correlates in independent cohorts (genetic pleiotropy).<br>• Factored in movement independent of exercise.<br>• Questionnaire administered face-to-face.<br>• HAPAQ questionnaire – validated for determination of levels of historical PA.<br>• Life calendar used to aid recall.	• Small cohort of C9-ALS patients.<br>• Link between gene expression changes in blood, and alterations within the CNS has not been evaluated.
Westeneng *et al.*, 2021[34]	Case-control study	To assess the causality of lifestyle factors during pre-symptomatic phase of ALS, stratified by *C9ORF72* mutation.	• Daily energy intake at symptom onset (C9+ group 712 kJ, 95% CI 212 to 1213, p=0·0053; C9- group 497, 295 to 700, p<0·0001) were higher in the C9+ and C9- groups.<br>• Combination of C9 gene and PA can increase risk of ALS.	• Recruited through neurologists and rehabilitation physicians in Netherlands, Dutch neuromuscular patient association and ALS centrum website – reduces selection bias.<br>• 50 years analysed pre-onset.<br>• They had three arms, one group with ALS and *C9ORF72* mutation, one group with ALS without the mutation and a control group – allowing a direct evaluation of whether a genetic-environmental effect is present.	• Questionnaires not validated.<br>• Case-control studies are prone to selection and recall bias and it is difficult to establish temporal causality, is the outcome a consequence of exposure or vice versa?<br>• Only 143 patients in C9+ group, difficult as rare population, but small numbers can reduce the power of the study.
Filippini *et al.*, 2020[41]	Case-control study	To assess non-genetic risk factors of ALS.	• Positive association of ALS risk with trauma (head) (OR=2·61, 95% CI 1·19-5·72), electric shock (OR=2·09, 95% CI 0·62-7·06), private wells for drinking water (OR=1·38, 95% CI 0·73-2·27), herbicides during gardening	• Controls chosen from Local Health Authority Registries (compulsory for Italians), limited selection bias.<br>• Recruitment from multiple locations: Emilia-Romagna Registry for ALS, Piemont and Valle d'Aosta Register for ALS, hospital discharge	• Excluded patients with *C90RF72* gene – did not assess potential combined genetic and environmental link.<br>• Small sample size due to a low response rate, affecting precision of the risk estimates.<br>• Self-report only – recall bias over a large time scale.

			(OR=0·27, 95% CI 0·12-0·6) and competitive sports.<br>• A slightly increased risk was associated with soccer at a competitive level (OR = 1·19, 95% IC 0·35–4·02), skiing (OR = 1·48, 95% CI 0·42–5·13), and marginally with swimming (OR = 1·61, 95% 0·09–27·33) due the very low number of exposed subjects.<br>• Any sport – inverse association (OR=0·77, 95% CI 0·41-1·42) including competitive.<br>• Slight difference in associations in different parts of Italy.	records, death certificate files and drug prescription directories.	• The report does not explain in the methods how they collected the data in detail, whether it was via a questionnaire and if so whether this was validated.<br>• The report does not discuss why these specific variables were chosen.
Canosa *et al.*, 2020[71]	Case-control study	To evaluate the metabolic correlates of lifetime sport practice in ALS through brain ^{18}F-FDG-PET	• In the group that did not undertake regular exercise significant hypermetabolism was found in areas known to be affected by ALS (frontotemporal regions and corticospinal tracts) despite same level of disability as expressed by ALS FRS-R.<br>• Patients who do not exercise regularly cope better with the neurodegenerative process in ALS.	• Multicentre study.<br>• Considered genetic risk-phenotype (*C90RF72*).<br>• Used the El Escorial criteria for diagnosis – standardised.<br>• Exposure to sports was measured as MET.<br>• Used metabolic consumption calculation and structured questionnaire with exposome aspects to limit recall bias.	• Only at diagnosis – may change before or later on in the disease process.<br>• The questionnaire is not detailed, and no statement that the questionnaire had been validated.
Korner *et al.*, 2019[35]	Cohort study	To assess potential risk factors to see if they affect onset and progression of ALS and whether specific risk factors are linked to distinct phenotypes of ALS.	• Patients did not report increased PA during leisure/occupation vs control.<br>• Positive correlation between increased extent of moderate training in ALS patients vs controls who tended to more frequently undergo training with higher intensity (not significant, p=0·0081).	• Asked via a personal interview developed by ONWeBDUALS, European ALS researchers.<br>• Considered distinct phenotypes and a wide range of potential risk factors.	• Retrospective interview-based – recall bias.<br>• Used spouses of patients for controls – gender difference and shared interests were not accounted for.<br>• Small cohort – did not allow for phenotype investigation.<br>• Patients were selected from one university hospital – selection bias.<br>• PA was graded intense PA for over one year or mild PA, - this is not very specific.<br>• The report does not state over how many years the questionnaire covered.
Venkataramni *et al.*, 2018[19]	Retrospective cohort study	Is regular participation as a player in the National Football League (NFL)	• There was no statistically significant difference in the risk of long-term all-cause mortality among career NFL players vs NFL replacement players who participated in the NFL during a 3-game league-wide player strike in 1987.	• Used Pro Football Reference - an online database covering the census of professional American football players since 1920s – validated and includes number of years and games played.	• There was no general control. NFL replacement players are likely to be healthy individuals who are constantly active – typically former college football players/players who were released from the NFL team during the pre-season/former players from rival leagues.

		associated with long-term mortality risk?			• Outcome was all-cause mortality – players may have developed ALS but not yet died of it.<br>• Excluded players who died prior to retirement from the NFL – ALS may have affected these figures as has short life expectancy, potentially earlier in physically active individuals.
Janssen *et al.*, 2017[18]	Cohort Study	To assess whether athletes who played American varsity high-school football (1956-1970) had an increased risk of neurodegenerative diseases later in life.	• Football players had an increased risk of medically documented trauma (especially if >1 year).<br>• Did not have increased risk of neurodegenerative disease overall.<br>• No cases of ALS in either 296 varsity football or 190 non-football varsity athletes.	• Reviewed entire medical record of each student – not solely hospital discharges.<br>• One researcher was blinded when assessing medical records – reduces bias.<br>• Compared diagnosis on notes with senior neurologist – no statistical differences.<br>• Long follow-up (median follow-up 64.64 years).	• Used male athletes as controls – swimmers, wrestlers, basketball players.<br>• Data were collected from year books, might not be accurate.<br>• No quantity of time or frequency of PA recorded, or position in the field.<br>• Head injuries were assessed by medical records – often under-reported.<br>• Only identified 75% of students in cohort and study population limited to the 30% who had prolonged medical records up to 2005 – selection bias.<br>• Study underpowered.
Fang *et al.*, 2016[40]	Case-control study	Do long distance cross-country skiers have a higher risk of ALS? And is the increased risk modified by skiing performance?	• Fastest skiers had >4x risk of ALS (HR 4·31, 95% CI 1·78-10.4).<br>• Skiers who participated >4 races during this period had higher risk (HR 3·13, 95% CI 1·37-7·17) vs 1 race.	• Controls recruited via Swedish Total Population Register and matched demographically – avoids selection bias.<br>• Conducted separate statistical comparisons with skiers and controls, due to skiers having other confounding variables.<br>• Adjusted for education, employment status, region of residence all obtained from national registries.<br>• Large scale 508,239 controls.<br>• Prospectively and independently collected data on exposure and outcome.<br>• Long-term and complete flow-up.	• ALS was identified by using hospital discharge letters, therefore patients just cared for in outpatients would not have been picked up.<br>• Not all variables accounted for, for example drug exposure; some skiers have higher Hb values.
Harwood *et al.*, 2016[27]	Case-control study	Examine any association between physical activity and the risk of	• An extra 10kJ/kg/day of PA (equivalent to 45 mins brisk walking) consistently associated with an increased risk ALS.<br>• Total PA throughout adulthood significant association with ALS (age adjusted OR 1·09, 95% CI 1·03-1·16, p=0·002).	• Use of a validated historical PA questionnaire – specifically designed.<br>• Adjusted for confounders: smoking, educational attainment.<br>• Identified from hospital and community ALS services – not just unwell patients.	• Small cohort of patients (n= 175 ALS versus 317 controls) – reduced power.<br>• Patients were excluded if family history of ALS/concurrent neurodegenerative disease.<br>• Data for the most recent 5 years were excluded to limit PA suppression – resulting from subclinical disease.<br>• It does not mention specific activities *e.g.*, soccer.

		developing ALS.		• Face-to-face interviews avoids recall bias – with life calendar of significant events to aid recall.<br>• Data included PA at home, work and in leisure.<br>• Used data from the most recent 15 years and whole of adulthood in the analysis.<br>• Participants were blinded to research hypothesis – therefore less over-estimation of activity levels due to being unaware of proposed association.<br>• Standardised data collection – structured manner at set time periods.<br>• Quantified energy expenditure of activities as ratio w/ standard resting metabolic rate, expressed as metabolic equivalents – related to oxygen consumption.	• Specific mutations *e.g.,* C9ORF72 not considered.<br>• Retrospective data collection – recall bias, over-estimate activity levels (and due to social desirability).<br>• HAPAQ validated with healthy controls, it is possible that ALS patients may recall PA differently.
Pasquinelli *et al.,* 2016[79]	Case-control study	Evaluate whether or not peroxisome proliferator-activated y coactivator 1a (PCG-1a) SNP 1444 G>A (Gly48Ser) could affect oxidative stress markers, both in basal conditions and during exercise fatigue testing in ALS patients.	• ALS patients with Gly482Ser allelic variant show increased exercise-related oxidative stress.<br>• Significantly increased advanced oxidation protein products (379·91+/- 206·97 nmol/ml; p<0·001), decreased ferric reducing ability (p<0·001) and thiol groups (p<0·001) in ALS vs controls.<br>• No relation between Gly482Ser polymorphism and oxidative stress biomarker levels were detected in resting conditions.<br>• Lactate levels were significantly higher (p<0·01 and p<0·001) and greater protein oxidative products were found in AA (Ser482Ser) compared to GG (Gly482Gly) and GA (Gly482Ser) ALS patients.<br>• sALS patients increased lactate levels at 30% (p<0·01), 50% (p<0·01) and 70% (p<0·001) of maximal voluntary contraction (MVC) vs baseline levels and at 70% (p<0·05) vs 30% of MVC.	• Analysed plasma levels of various oxidative stress biomarkers.<br>• Looked at incremental times connected to a myometer – checking contractile force, at baseline, end of each step and 15 min after exercise – aerobic at the beginning and anaerobic with increased force – quantified results.	• All participants recruited at the Department of Clinical and Experimental Medicine, Neurological Clinic – selection bias.<br>• Small sample 197 recruited, only 35 tested for antioxidant activity (total SOD and catalase) and 28 oxidative stress markers during exercise (AOOP, FRAP, thiols and lactate).<br>• Exercise exposure was only for 22 minutes, does not account for long-term effects or repetitive strenuous physical activity.
Lehman *et al.,* 2012[47]	Cohort study	Is there an increase in deaths from neurodegenerative causes	• Overall mortality vs US population reduced.<br>• Neurodegenerative mortality increased – ALS and AD 4x.<br>• Higher neurodegenerative mortality in speed positions (considered to have a higher	• Large sample size (3439 football players).<br>• Death certificates were coded by a certified Nosologist when information was lacking from the National Death Index.	• Classified into speed and non-speed players to assess for high acceleration head impacts – this does not quantify head impact, just estimates due to position in the field.

		(AD, PD, ALS) in professional football players vs general population?	incidence of concussion) vs players in non-speed positions.	• Additional analyses looking at multiple cause of death (MCOD) rate files to examine all causes, as neurodegenerative disorders commonly listed as contributing cause, not main cause of death.	
Savica *et al.,* 2012[16]	Cohort study	To assess whether high school football players 1946-1956, (when headgear was less protective than today), was associated with development of neurodegenerative diseases later in life.	• No increased risk of dementia, PD or ALS.<br>• Neuropathological examination of an ALS football player, showed no pathological findings supportive of CTE.	• Reviewed all medical records to assess for diagnoses, as opposed to hospital records only.	• Controls were 'non-football' playing, it does not specify if they played other sports or the activity levels of either group.<br>• Follow-up 50 years after high school graduation may not extend sufficiently to allow inclusion of all neurodegenerative diagnoses.<br>• 35% of controls had no medical visits or left the country so were excluded – selection bias.<br>• Severity of concussions was speculative.<br>• Outcome rare – only 2 cases of ALS in the football group and 1 in non-football group.<br>• 438 football players vs 140 controls.<br>• Restricted to one area – Rochester, Minnesota.
Mattsson *et al.,* 2012[15]	Cohort study and matched case-control study	To assess if physical fitness is implicated in the aetiology of ALS and subsequent death	• Weight adjusted physical fitness (W/kg) (OR 1·81 (95% CI 1·07 to 3·06)) but not physical fitness per se, was a risk factor for ALS.<br>• The higher the ability of men to move their own body weight, the greater the risk of subsequent death from ALS.<br>• Resting HR was numerically higher in cases than in controls (mean 77 vs 74.6 p=0·09).	• Large sample size 684,459, covered whole young male population of a country – low dropout rates.<br>• Used Swedish military service conscription examination at age of 18, highly standardised.<br>• Used maximum load that conscript could sustain for 6 minutes (WMAX6 min) – good reliability (correlation coefficient 0.90) and correlates well with other endurance tests. [35]<br>• Used ALS death certificates and validated against El-Escorial criteria in medical records.<br>• Measures on anthropometrics and physical capacities were highly standardised and not biased by self-reporting.	• 7% incorrect diagnoses in the validation set – (in line with false positive diagnosis of 8% in previous study).<br>• Did not evaluate differences in life course PA between cases and controls.<br>• On average heavy men started on higher loads than slimmer men did – reduced their chance of obtaining low values – underestimated the physical fitness in men who later developed ALS.<br>• Other confounding variables were not measured – genetics, smoking, toxin exposure etc.

Turner *et al.*, 2010[33]	Cohort study	Does handedness influence the side of upper-limb onset disease and footedness in lower limb-onset ALS, highlighting a link between PA and ALS?	• For upper limb-onset patients there was concordance for side of onset and handedness (97/151 or 64%, p=<0·0006).<br>• Lower limb-onset concordance for side of onset and footedness was absent (99/181 or 55% p=0·234).<br>• Exercise influences pathogenesis of ALS as routine physical demands on upper limb are heavily influenced by limb dominance, in lower limbs commonest function is standing/locomotion which places similar PA demands on both limbs.	• The specific hypothesis was not mentioned to participants (blinded) and only able to submit one questionnaire – reduce bias.<br>• 502 patients responded (high response rate).	• ALS patients registered with internet-based support site were chosen to participate – selection bias.<br>• Small sample size.<br>• Recall bias and responder bias due to nature of questionnaire.<br>• Recall bias – likely weakness will be noticed sooner in a dominant limb if both limbs are equally weak, due of greater awareness and higher use.
Chio *et al.*, 2009[14]	Retrospective cohort study	To assess the risk of ALS in two different cohorts of Italian professional athletes (road cyclists and basketball players) vs soccer players.	• Highly significant risk of developing ALS in soccer players, with young age of onset, dose-effect risk and predilection for midfielders.<br>• No apparent increased risk of ALS in road cyclists and basketball players – concluded that ALS may not be related to physical activity per se.	• Longitudinal study, original study 1970-2001, extended to 2006.	• No detail on quantifying the amount of exercise.<br>• The questionnaire-based quantification of exercise allows for recall and confounding bias.<br>• Failure to consider risk-phenotypes.<br>• Basketball is mainly anaerobic, characterised by VO_{2MAX} lower than that of soccer players.<br>• Road cyclists have a higher VO_{2MAX} than soccer players.<br>• Small cohort sizes for the different sporting activities.
